# A tale of two cities: the cost, price-differential and affordability of current and healthy diets in Sydney and Canberra, Australia

**DOI:** 10.1186/s12966-020-00981-0

**Published:** 2020-06-22

**Authors:** Amanda J. Lee, Sarah Kane, Lisa-Maree Herron, Misa Matsuyama, Meron Lewis

**Affiliations:** 1 School of Public Health, The University of Queensland, 288 Herston Road, Herston, QLD 4006 Australia; 2 Queensland University of Technology, Victoria Park Road, Kelvin Grove, QLD 4059 Australia

**Keywords:** Diet price, Food price, Food affordability, INFORMAS, Nutrition policy, Fiscal policy

## Abstract

**Background:**

The perception that healthy foods are more expensive than unhealthy foods has been reported widely to be a key barrier to healthy eating. However, assessment of the relative cost of healthy and unhealthy foods and diets is fraught methodologically. Standardised approaches to produce reliable data on the cost of total diets and different dietary patterns, rather than selected foods, are lacking globally to inform policy and practice.

**Methods:**

This paper reports the first application, in randomly selected statistical areas stratified by socio-economic status in two Australian cities, of the Healthy Diets Australian Standardized Affordability and Pricing (ASAP) method protocols: diet pricing tools based on national nutrition survey data and dietary guidelines; store sampling and location; determination of household incomes; food price data collection; and analysis and reporting. The methods were developed by the International Network on Food and Obesity/NCD Research, Monitoring and Action Support (INFORMAS) as a prototype of an optimum approach to assess, compare and monitor the cost and affordability of diets across different geographical and socio-economic settings and times.

**Results:**

Under current tax policy in Australia, healthy diets would be 15–17% less expensive than current (unhealthy) diets in all locations assessed. Nevertheless, healthy diets are likely to be unaffordable for low income households, costing more than 30% of disposable income in both cities surveyed. Households spent around 58% of their food budget on unhealthy food and drinks. Food costs were on average 4% higher in Canberra than Sydney, and tended to be higher in high socioeconomic locations.

**Conclusions:**

Health and fiscal policy actions to increase affordability of healthy diets for low income households are required urgently. Also, there is a need to counter perceptions that current, unhealthy diets must be less expensive than healthy diets. The Healthy Diets ASAP methods could be adapted to assess the cost and affordability of healthy and unhealthy diets elsewhere.

## Background

Suboptimal diet is the leading preventable risk factor contributing to non-communicable disease (NCD) globally [1], and in Australia [2]. Less than 4% of the Australian population consume diets consistent with Dietary Guidelines [1, 3, 4]. In Australia 63% of adults and 25% of children aged two to 17 years are overweight or obese [5]. More than one-third of adults’ and children’s energy intake (35 and 39% respectively) comes from unhealthy, ‘discretionary’ sources, defined as foods and drinks that are not required for health that are high in saturated fats, added sugar, salt and/or alcohol [4, 6].

Dietary behaviors are affected by a wide range of influences, including the price, availability, accessibility and promotion of foods [7]. The public perception that healthy foods are expensive has been reported widely as a barrier to healthy diets, particularly in low socioeconomic groups [8], and an important contributor to diet-related health inequities in Australia [9–13]. Comprehensive monitoring of food environments is essential to understand, and support policy action to address, environmental factors that influence dietary choices and, in turn, the burden of disease [14]. In Australia, historical monitoring of food environments has had a strong focus on the cost of foods, with ‘food basket’ methods used to assess the cost of a ‘healthy’ diet in different locations and over time [15]. However, results are not comparable, as over six different approaches have been used, with variation in the contents of a ‘healthy’ basket, reference household composition, source of household income, sampling methods, seasonality and data collection protocols [15, 16]. Further, the majority of diet pricing tools included commonly consumed ‘unhealthy’ foods (such as sugar, sausages, soft drinks, meat pies and chocolate) in the ‘healthy’ basket, hence did not fully align with current Australian Dietary Guidelines [4, 15]. For over 30 years, such studies have confirmed that food prices increase over time, and that food prices in rural and remote areas are at least 30% higher than those in capital cities in Australia [15]. To date, no national survey of food or diet prices from a health perspective has been conducted, and the available state, regional and local data have not been used to improve policy or practice.

A range of complex determinants influence food prices, such as political, economic, sociocultural and environmental factors [8]. Governments have the potential to manipulate food pricing through fiscal policies (taxation and/or subsidies) to improve population diets and reduce the rates of obesity and NCDs [8, 17]. In Australia, differential taxation has been applied since 2000: basic healthy foods are exempt from Goods and Services Tax (GST), while all other foods and drinks are subject to 10% GST [18].

To inform policy action on food pricing and affordability, robust and reliable data are urgently needed [15]. The International Network on Food and Obesity/NCD Research, Monitoring and Action Support (INFORMAS) has developed a step-wise framework to determine the relative price and affordability of different diets. The INFORMAS framework’s ‘optimal’ approach assesses the cost, price differential and affordability of healthy (dietary guideline recommended) diets, and current diets based on reported intake in national surveys [8]. Given the high rates of poor-diet related outcomes in Australia, current reported diets can be considered unhealthy [1]. Based on the ‘optimal’ INFORMAS approach, a standardized method to assess and compare the cost and affordability of recommended and reported diets – the Healthy Diets ASAP (Australian Standardised Affordability and Pricing) protocol – was developed as a prototype in Australia [19, 20].

This paper reports the first application of the Healthy Diets ASAP methods in two major cities in Australia: Canberra (the national capital) and Sydney (the most populous city). These cities were surveyed at the requests of the respective state/territory Health Departments. The road distance between the two cities is approximately 300 km. Based on 2016 census data, the population of Canberra was 196,037 (50.8% female) with a median age of 37 years [21]. More than one-third of residents (35.7%) reported having completed a Bachelor Degree or above. Of people aged 15 years and over, 63.5% worked full-time; a large proportion (over 30%) in the public sector (i.e. in government departmental administration). Rented dwellings comprised 29.7% of occupied residences, with a median rent of A$390 per week.

The 2016 census data for the Greater Sydney area [22] reported a total population of 4,823,991; 50.7% were female, and the median age was 36 years. Of people aged 15 years and over, 28.3% reported having completed a Bachelor Degree level or above; and 61.2% worked full-time. The top five industries of employment were hospitals; computer system design and related services; cafes and restaurants; banking; and supermarket and grocery stores. Around one-third (34.1%) of occupied dwellings were rented, with a median weekly rent of A$440.

This study assessed and compared the price, price differential (relative price) and affordability of healthy (recommended) and reported current (unhealthy) diets for a reference family of four in randomly sampled locations in different socioeconomic quintile areas in Sydney and Canberra.

## Methods

The Healthy Diets ASAP methods protocol was developed collaboratively [19, 20] and applied as described in detail elsewhere [20]. The complex protocol comprises five parts: standardized healthy and current diet pricing tools; store location and sampling; calculation of median and low-income household incomes; price data collection; and analysis and reporting.

### Diet pricing tools

The pricing tools for both the current and healthy diets include quantities of food for a fortnight for a reference household of a family of four, comprising an adult male 31–50 years old, an adult female 31–50 years old, a 14 year old boy and an 8 year old girl, as per the protocol [20]. The current diet pricing tool is based on food and drink intake reported in the Australian Health Survey (AHS) 2011–12 [5]. It tallies the mean intake of foods and drinks for each age and gender group of the four individuals in the reference household. The healthy diet pricing tool contains types and quantities of food and drinks consistent with the recommendations of the *Australian Dietary Guidelines* and the *Australian Guide to Healthy Eating* [4, 23]. Consistent with recommendations and expert stakeholder advice [20], the healthy diet pricing tool includes choices from the five food groups and no discretionary choices. A summary of the included food and drinks is provided in Table 1. Details of the diet pricing tools including quantities are in Supplementary information file 1.

**Table 1:** Foods and drinks included in the Healthy Diets ASAP (Australian Standardised Affordability and Pricing) protocol^a^

Healthy Diets ASAP diet component	Foods and drinks
Healthy Diet	**● Water** (bottled) **● Fruit**: apples, bananas, oranges **● Vegetables:** potatoes, broccoli, white cabbage, iceberg lettuce, onion, carrot, pumpkin, tomatoes, sweetcorn (canned), four bean mix (canned), diced tomatoes (canned), baked beans (canned), frozen mixed vegetables, frozen peas, salad vegetables in sandwich **● Grain (cereals):** wholegrain cereal biscuits (Weet-bix™), rolled oats, cornflakes, wholemeal bread, white bread, white rice, white pasta, dry water cracker biscuit, bread in sandwich **● Lean meats and alternatives:** beef mince and steak, lamb chops, cooked chicken, tuna (canned), eggs, peanuts (unsalted), meat in sandwich **● Milk, yoghurt and cheese:** cheddar cheese (full fat, reduced fat), milk (full fat, reduced fat), yoghurt (full fat plain, reduced fat flavoured) **● Unsaturated oils and spreads:** olive oil, sunflower oil, canola (margarine)
Unhealthy Diet	**● Healthy foods** as above; in reduced amounts **● Drinks:** artificially sweetened soft drink, sugar sweetened soft drink, orange juice **● Cereals, snacks and desserts:** muffin, sweet biscuits, savoury biscuits, confectionary, chocolate, potato crisps, muesli bar, peanuts (salted), ice cream, fruit salad (canned in juice) **● Processed meats:** beef sausages, ham **● Spreads, sauces, condiments and ingredients:** butter, tomato sauce, salad dressing, white sugar **● Convenience meals:** frozen lasagne, chicken soup (canned), fish fillet (crumbed), instant noodles, meat and vegetable stew (canned) **● Fast food:** pizza, meat pie, hamburger, potato chips/fries **● Alcohol:** beer (full strength), white wine (sparkling), red wine, whisky

^a^Table adapted from the Healthy Diets ASAP protocol developed by Lee et al. [19, 20]

### Store location and sampling

A random sample of locations in each city was identified according to the Healthy Diets ASAP Protocol [20]. The Statistical Area Level 2 (SA2) locations (medium-sized geographical areas, generally with a population of 3000 to 25,000 [24]) in each city were stratified by Socio-Economic Indexes for Areas (SEIFA) quintile [24]. The SEIFA Index of Relative Socioeconomic Disadvantage (IRSD) ranks areas in Australia according to relative socio-economic disadvantage based on information from the five-yearly national Census data. Two SA2 locations within each of the SEIFA quintiles 1, 3 and 5 were selected randomly. As per the protocol, for each location, Google Maps [25] was used to identify food outlets within 7 km by car from the geographical centre [20]; stores surveyed included one outlet of each supermarket chain and independent grocer (Coles®, Independent Grocers Australia (IGA®), Supabarn® and Woolworths®), prescribed ‘fast food’/takeaway outlets of most commonly consumed ‘fast foods’ (a Big Mac® burger from a McDonald’s® restaurant; pizza from Pizza Hut®; hot chips from an independent fish and chips store), and two alcoholic beverage stores.

### Price data collection

An experienced Research Assistant in each city was trained in the application of the Healthy Diets ASAP survey form to collect food and drink prices strictly according to the published data collection protocol [20]. For example, the data collection sheet specifies common brands and sizes and what to do if the specified product is not available or is on price promotion. AL moderated 10% of data collection points; concordance was high at over 98%, and was deemed acceptable. Food prices were collected between 4 November 2015 and 5 December 2015. Permission to collect food prices was sought from each store manager immediately prior to data collection. Only one manager declined; another store of the same chain next closest to the geographical centre of the area was surveyed.

### Calculation of household incomes

Median gross household income per week (before taxation, rent and other expenses) in the six selected locations was calculated according to the Healthy Diets ASAP protocol [20]. The reported median weekly gross household income in Canberra was A$2498 [21] and A$1988 in Sydney [22]. This was adjusted for a wage price index increase of 11.1% from September 2011 to September 2015, and multiplied by two to derive the median gross household income in each area per fortnight [26]. Detailed data are presented in Supplementary information file 2.

The indicative low income of each household was calculated according to the Healthy Diets ASAP protocol [20]. Adjustments were made for the taxation payable by members of the representative household to derive this metric. Detailed household income data are presented in Supplementary information file 3.

### Analysis and reporting

Data from the completed price data survey forms were double entered into Microsoft® Office Excel (2016) spreadsheets, checked and cleaned (by SK and ML). One missing value was ascribed the mean price of the same item in other supermarkets in the same SA2 area in the same city. The mean price of the current and healthy diet for the representative household was calculated for each SEIFA quintile in each city. Other metrics calculated included the cost of purchasing foods from the recommended five food groups and healthy oils and spreads allowance [4] and the cost and proportion of household food budget spent on discretionary items including alcoholic drinks, takeaway foods and sugar-sweetened beverages.

Affordability of each diet was determined according to the Healthy Diets ASAP protocol [20], calculated as the proportion of both the median gross household income and the indicative low disposable income of the reference households required to purchase the current and healthy diets. Diet affordability was deemed unacceptable if it cost more than 30% of household income [27]; food stress was deemed to occur when a household needed to spend more than 25% of their disposable income on food [28, 29].

## Results

### Cost of the different diets in Sydney and Canberra

Food prices were collected in 12 geographical areas, six each in Sydney and Canberra. The total costs of the current and healthy diets for the reference household per fortnight are presented for each of the SEIFA quintiles in Sydney and Canberra in Tables 2 and 3.

**Table 2:** Cost of the current and healthy diets in SA2 locations, Sydney, per household per fortnight

SEIFA Quintile	Quintile 1 (most disadvantaged)						Quintile 3						Quintile 5 (least disadvantaged)					
Diet type	Current (unhealthy)			Healthy (recommended)			Current (unhealthy)			Healthy (recommended)			Current (unhealthy)			Healthy (recommended)		
	Mean cost (A$)	SD (A$)	Prop of total (%)	Mean cost (A$)	SD (A$)	Prop of total (%)	Mean cost (A$)	SD (A$)	Prop of total (%)	Mean cost (A$)	SD (A$)	Prop of total (%)	Mean cost (A$)	SD (A$)	Prop of total (%)	Mean cost (A$)	SD (A$)	Prop of total (%)
**Water, bottled**	15.35	5.23	2	15.35	5.23	3	13.88	4.31	2	13.88	4.31	2	16.54	4.93	2	16.54	4.93	3
**Fruit**	47.78	8.18	7	55.63	19.75	9	53.64	3.64	7	72.10	5.54	12	54.71	4.97	7	71.98	9.95	12
**Vege (& legumes)**	40.52	3.17	6	99.92	9.74	17	40.46	4.01	6	102.85	9.56	17	42.74	4.97	6	108.21	11.79	17
**Grain (cereal) foods**	46.50	5.22	7	119.60	21.02	20	44.50	3.25	6	110.73	12.91	18	45.07	3.94	6	107.13	13.62	17
**Meats, poultry, fish, eggs, nuts & seeds**	95.37	3.94	14	179.69	6.14	31	93.15	6.31	13	183.41	13.36	31	98.54	6.32	13	189.12	14.83	31
**Milk, yoghurt, cheese & alternatives**	45.24	1.83	6	109.52	3.56	19	45.74	2.28	6	108.53	5.15	18	48.87	3.27	6	116.81	6.03	19
**Unsaturated oils and spreads**	1.36	0.24	0	9.04	0.58	2	1.27	0.01	0	8.97	0.49	1	1.28	0.03	0	8.90	0.36	1
**Artificially sweetened soft drink**	5.15	0.46	1				5.16	0.44	1				5.37	0.67	1			
**All other discretionary choices**	407.17	16.50	58				431.68	17.55	59				441.76	25.27	59			
**Total**	**704.44**	**24.93**	**100**	**588.75**	**45.81**	**100**	**729.48**	**22.45**	**100**	**600.47**	**30.60**	**100**	**754.88**	**49.39**	**100**	**618.69**	**36.99**	**100**
**Costs of different components of diet**																		
***Core food***																		
**Combined fruit & vegetables**	88.3	10.66	13	155.54	27.90	26	94.10	5.88	13	174.95	13.45	29	97.44	7.23	13	180.20	15.79	29
***Discretionary food***																		
**Alcoholic drinks**	83.10	1.85	12				86.12	4.16	12				83.42	1.50	11			
**Takeaway foods**	121.86	9.10	17				133.14	8.92	18				138.92	5.22	18			
**Sugar sweetened beverages**	25.88	2.32	4				25.95	2.21	4				26.99	3.35	4

**Table 3:** Cost of the current and healthy diets in SA2 locations, Canberra, per household per fortnight

SEIFA Quintile	Quintile 1 (most disadvantaged)						Quintile 3						Quintile 5 (least disadvantaged)					
Diet type	Current (unhealthy)			Healthy (recommended)			Current (unhealthy)			Healthy (recommended)			Current (unhealthy)			Healthy (recommended)		
	Mean cost (A$)	SD (A$)	Prop of total (%)	Mean cost (A$)	SD (A$)	Prop of total (%)	Mean cost (A$)	SD (A$)	Prop of total (%)	Mean cost (A$)	SD (A$)	Prop of total (%)	Mean cost (A$)	SD (A$)	Prop of total (%)	Mean cost (A$)	SD (A$)	Prop of total (%)
**Water, bottled**	19.56	5.24	3	19.56	5.24	3	19.78	1.40	3	19.78	1.40	3	21.31	2.74	3	21.31	2.74	3
**Fruit**	57.27	3.11	8	72.65	7.33	12	57.49	5.26	8	75.93	7.99	12	60.57	9.57	8	81.86	9.55	13
**Vege (& legumes)**	40.40	0.89	5	103.45	3.96	17	40.06	4.35	5	103.38	10.63	17	40.91	4.22	5	105.26	13.50	17
**Grain (cereal) foods**	47.58	4.68	6	114.53	11.39	18	47.17	5.48	6	114.58	12.36	18	47.01	5.44	6	112.46	12.13	18
**Meats, poultry, fish, eggs, nuts & seeds**	95.01	3.62	12	186.32	3.9	30	97.28	5.52	13	190.11	13.29	30	95.37	10.37	12	185.90	1.18	30
**Milk, yoghurt, cheese & alternatives**	52.26	4.36	7	120.10	11.1	19	49.26	3.01	7	113.34	3.65	18	49.54	2.22	6	113.35	5.87	18
**Unsaturated oils and spreads**	1.25	0.32	0	9.01	0.31	1		0.19	0	8.71	0.66	1	1.25	0.17	0	9.23	0.75	1
**Artificially sweetened soft drink**	5.49	0.38	1				5.35	0.01	1				5.48	0.37	1			
**All other discretionary choices**	444.08	27.6	58				435.22	36.31	58				446.51	30.97	58			
**Total**	**762.90**	**36.6**	**100**	**625.62**	**16.53**	**100**	**752.78**	**48.47**	**100**	**625.83**	**36.26**	**100**	**767.95**	**53.74**	**100**	**629.37**	**49.39**	**100**
**Costs of different components of diet**																		
***Core food***																		
**Combined fruit & vegetables**	97.67	3.14	13	176.11	10.65	28	97.55	8.07	13	179.31	14.67	29	101.48	13.09	13	187.13	21.38	30
***Discretionary food***																		
**Alcoholic drinks**	89.31	5.54	12				87.41	2.35	12				91.38	4.80	12			
**Takeaway foods**	126.28	5.29	17				119.06	0.97	16				126.37	3.92	16			
**Sugar sweetened beverages**	27.57	1.91	4				26.86	0.05	4				27.54	1.84	4			

Across the included SA2 locations in SEIFA quintiles 1, 3 and 5, the mean ± standard deviation cost of the current diet across all SEIFA was A$729.60 ± 34.47 in Sydney and A$761.21 ± 46.82 in Canberra. The mean cost of the recommended (healthy) diet was A$602.64 ± 20.07 in Sydney and A$626.94 ± 36.64 in Canberra. Therefore the cost of both diets was approximately 4% more in Canberra than Sydney. Compared to Sydney prices, in Canberra the mean costs of the current and recommended (healthy) diets were both 2% more in the least disadvantaged areas, but 8 and 6% more respectively in the most disadvantaged areas.

In both cities, it would cost the representative household 17–18% less to purchase the healthy diet than the current diet.

Across all locations in both cities, approximately 58% of the household food budget would be expended on discretionary food and drinks, in purchasing the types and amounts reported in the current diet. In both Sydney and Canberra, and across quintiles, households purchasing the current diet would spend an average of around 17% of the food budget on takeaway foods, 12% on alcoholic drinks, and 4% on sugar-sweetened drinks (Table 2 and 3).

### Differences in the cost of diets across SEIFA quintiles

In Sydney, the cost of each diet increased linearly across the SEIFA quintiles. The current diet was 7% more expensive in the least disadvantaged area (A$754.88 ± 49.39) than the most disadvantaged area (A$704.44 ± 24.93); the cost of the healthy diet would be 5% more expensive in the least disadvantaged area (A$618.69 ± 36.99) than the most disadvantaged area (A$588.75 ± 45.81) (Table 2). Therefore, in Sydney the healthy diet would cost approximately 16% less than the current diet in the most disadvantaged areas, but the difference in cost would be 18% in the least disadvantaged areas (Table 2).

In contrast, in Canberra, the cost of both the current and healthy diets varied by only 1–2% across all SEIFA Quintile areas surveyed. While the cost of both diets was highest in the least disadvantaged area (A$767.95 ± 53.74 and A$629.37 ± 49.39 respectively), the current diet was slightly less expensive in the median quintile than in the most disadvantaged quintile, and the cost of the healthy diet would be very similar in these locations (Table 3). In Canberra, the healthy diet would be 18% less expensive than the current diet in both the most and the least disadvantaged areas, but the difference was slightly less (17%) in the areas of median quintile of disadvantage (Table 3).

Across all locations, only approximately 13% of the household food budget would be spent on fruit and vegetables in the current diet; by comparison, more than one-quarter of the household food budget (26–30%) would be required to meet the recommended intake of fruit and vegetables (Table 2 and 3). Similarly, across all locations, a household purchasing the current diet would expend less than half – and in several cases less than one-third – of the proportions of the food budget required to meet the recommended intakes of the other healthy food groups including grain (cereal) foods, protein sources (meat, poultry, fish, eggs, nuts and seeds) and dairy foods or alternatives (Table 2 and 3).

A similar proportion (around 17%) of the food budget was spent on average on takeaway foods in both cities. However, in Sydney, takeaway foods cost 12% more in the least disadvantaged areas than the most disadvantaged areas, while in Canberra, takeaway foods cost 6% more in both the most and least disadvantaged areas compared to areas of median SEIFA quintile (Table 3).

The prices of alcoholic and sugar-sweetened beverages were similar across all SA2 areas in both cities, varying by only 3–4%. The mean price of the alcoholic drinks reported in current diets was A$84.21 ± 1.66 in Sydney and A$89.37 ± 1.99 in Canberra. It would cost approximately 12% of the total household food budget to purchase the types and volumes of alcoholic beverages reported in the AHS 2011–12 across all SA2 areas in both cities. The mean cost of sugar sweetened beverages in the current diet was A$26.27 ± 0.62 and A$27.32 ± 0.40 in Sydney and Canberra respectively, requiring around 4% of the household food budget in all areas surveyed.

### Income and affordability of different diets

Affordability of the healthy and current diets was calculated using both median and indicative low disposable incomes of the reference household per fortnight (Table 4).

**Table 4:** Median gross, and indicative low disposable household income, and affordability of different diets in Sydney and Canberra

City	Sydney														
SEIFA Quintile	Quintile 1 (most disadvantaged)					Quintile 3					Quintile 5 (least disadvantaged)				
**Diet type**		**Current (unhealthy) diet**		**Healthy (recommended) diet**			**Current (unhealthy) diet**		**Healthy (recommended) diet**			**Current (unhealthy) diet**		**Healthy (recommended) diet**	
	**Income A$**	**Cost A$**	**Affordability (% income)**	**Cost A$**	**Affordability (% income)**	**Income A$**	**Cost A$**	**Affordability (% income)**	**Cost A$**	**Affordability (% income)**	**Income A$**	**Cost A$**	**Affordability (% income)**	**Cost A$**	**Affordability (% income)**
**Median gross household income**^**a**^	1868.00	704.44	38%	588.75	32%	3094.00	729.48	24%	600.47	19%	4528.00	754.88	17%	618.69	14%
**Low household disposable income**^**b**^	2234.68	704.44	32%	588.75	26%	2234.68	729.48	33%	600.47	27%	2234.68	754.88	34%	618.69	28%
City	Canberra														
SEIFA Quintile	Quintile 1 (most disadvantaged)					Quintile 3					Quintile 5 (least disadvantaged)				
**Diet type**		**Current (unhealthy) diet**		**Healthy (recommended) diet**			**Current (unhealthy) diet**		**Healthy (recommended) diet**			**Current (unhealthy) diet**		**Healthy (recommended) diet**	
	**Income A$**	**Cost A$**	**Affordability (% income)**	**Cost A$**	**Affordability (% income)**	**Income A$**	**Cost A$**	**Affordability (% income)**	**Cost A$**	**Affordability (% income)**	**Income A$**	**Cost A$**	**Affordability (% income)**	**Cost A$**	**Affordability (% income)**
**Median gross household income**^**a**^	3562.00	762.90	21%	625.62	18%	4412.00	752.78	17%	625.83	14%	6130.00	767.95	13%	629.37	10%
**Low household disposable income**^**b**^	2234.68	762.90	34%	625.62	28%	2234.68	752.78	34%	625.83	28%	2234.68	767.95	34%	629.37	28%

^a^Median gross household income – the median income of a household within the SA2 areas surveyed for this quintile, sourced from ABS Community profiles

^b^Indicative low household disposable income – the income determined for this household, including all appropriate payments and supplements, sourced from Department of Human Services website calculators. This calculation is not location dependent

Median gross household income per fortnight varied greatly across the SEIFA quintiles in both cities. In Sydney, the median household income ranged from A$1868 in Quintile 1 to A$4528 in Quintile 5. In Canberra, median household incomes were much higher than in Sydney in all SA2 areas, ranging from A$3562 in Quintile 1 to A$6130 in Quintile 5. The median income of the most disadvantaged areas in Canberra was almost 50% higher than that in Sydney.

Affordability of both current and healthy diets was lowest in the most disadvantaged areas in both cities. However, in these areas affordability was markedly lower in Sydney; current and healthy diets would cost respectively 38 and 32% of median household income in Sydney and respectively 21 and 18% of median household income in Canberra.

Indicative low disposable income of the representative household per fortnight was based on national data, therefore it was the same across all locations (A$2234.68). It was lower than the median household incomes in all areas in Canberra, but higher than the median household income in the most disadvantaged area (Quintile 1) in Sydney. Therefore, both current and healthy diets were much less affordable when assessed against indicative low household income than median gross household income in all locations assessed except SEIFA Quintile 1 areas of Sydney.

In the areas of least disadvantage, the affordability of both diets assessed against indicative low household income was at least half affordability assessed against median gross household income. In Sydney, the current diet cost 32–34% and the healthy diet cost 26–28% of the indicative low household income. In Canberra, the current (unhealthy diet) cost 34% and the healthy (recommended diet) diet cost 28% of the indicative low household income in all areas.

Overall, compared with both median gross and indicative low disposable household incomes, the healthy diet was more affordable than the current diet in both cities and all areas assessed.

## Discussion

Previously, there has not been a standardized method to assess the cost and affordability of different diets from a health perspective across different geographical locations or times [8, 15, 16]. Application of the standardized Healthy Diets ASAP method enabled assessment and comparison of the cost, price differentials and affordability of current and healthy diets in two major Australian cities.

### Healthy diets can be less expensive than current diets

This study found that healthy diets would be less expensive than the current diets reported in the AHS 2011–12, in both Sydney and Canberra and across all SA2 locations stratified by SEIFA quintiles. These results accord with results of previous testing of the approach [19, 20] and application of the Healthy Diets ASAP method in rural towns of Victoria [30], further challenging the popular assumption that healthy foods and diets are always more expensive than unhealthy foods and diets [8, 12, 31, 32].

There are many methodological challenges in comparing the cost and affordability of healthy and unhealthy foods and diets [8, 33] including the selection, number and relationship of foods for pricing; the composition of the reference household; the consideration of quality and/or availability measures [34, 35]; sources of household income; store sampling frameworks; season of data collection; data collection protocols; and analysis methods. Specific methodological problems when focussing on comparison of the costs of selected foods rather than diets include: the lack of rationale to compare foods across different product categories; difficulties in comparing foods of different weights, volumes and energy densities; the different results obtained depending on which unit (energy, weight or portion) is used to present results [36], and lack of an ‘anchor’ determining the numbers of foods included in pricing lists [8]. Many papers examine the energy density and energy cost of foods [37], or of diets [38–40], however such approaches are mathematically spurious as they show a relationship between metrics that both include the same unit (i.e. ‘energy’) [36, 41].

However, the optimal approach to assess food costs from a health perspective outlined by INFORMAS addresses these methodological challenges [8], addressing the issues raised in a systematic review of diet pricing methods used previously in Australia [15]. As a prototype of this optimal approach, the Healthy Diets ASAP protocol provides a robust method to assess, benchmark and monitor the cost, price differential and affordability of healthy (recommended) diets and current (unhealthy) diets nationally [19, 20].

A systematic review and meta-analysis that found that healthy diets tend to cost slightly more than unhealthy diets [31] did not include Australian studies (Australia is one of only a few countries that applies a differential tax to foods based on health criteria). Furthermore, the costs of commonly consumed unhealthy items, such as alcohol and takeaway foods, were not reported consistently in the included studies. Hence it is not so surprising that, in Australia, healthy diets have been found to be less expensive than unhealthy diets that include such items.

### Most of the food budget is spent on unhealthy foods and drinks

More worryingly, this study identified that a reference family of two adults and two children in Sydney and Canberra in 2015 would spend 58% of their household food budget on unhealthy, discretionary items (Tables 2 and 3), which provided around 38% of their energy intake [22]. Such results are closely in line with Australian Dietary Guidelines Price Index data [42], which show discretionary food items accounted for 58.2% of household food expenditure in 2014. This study found a high proportion of the food budget would be spent on alcoholic drinks (approximately 12%), unhealthy takeaway foods (around 17%) and sugary drinks (4%).

These findings confirm that factors other than food price, such as convenience, ‘comfort’ and/or desirability and ‘taste’ (both physiological and economic), and the determinants of these factors (e.g. the ubiquitous availability, advertising and marketing of discretionary choices, poor food literacy, low cooking skills and busy lifestyles), may have greater impact on food choice [4, 19, 43].

### Both current and healthy diets are unaffordable for low income households in some areas

To be meaningful, measures of diet costs need to relate to income or purchasing power [44]. An arbitrary benchmark of 30% of disposable household income was applied as an indicator of diet affordability; households spending more than 25% of their income were considered at risk of food stress [27–29]. Disposable median household income data are not readily available in Australia, and median gross household income, which varies across different SA2 areas, is often used as a proxy [45], although this exaggerates estimates of affordability.

Nevertheless, this study found that in Sydney, even using median gross household income, both current and healthy diets were much less affordable in the most disadvantaged areas: affordability exceeded both food stress and affordability benchmarks at 38% for current diets and 32% for healthy diets. Hence families living in the most disadvantaged areas of Sydney are highly likely to be suffering from food insecurity.

In all other areas in Sydney and Canberra, when assessed against median gross household income, affordability appeared acceptable, with both diets costing only 10–17% of median gross household income in the less disadvantaged areas. While households in the most disadvantaged area of Canberra still needed to commit a higher proportion of their median gross income to purchasing food than those in other areas, affordability was attenuated due to the higher median gross household incomes in all areas of Canberra compared to Sydney. For example, in SEIFA Quintile 1 median gross household income in Canberra was almost double that in the same SEIFA quintile in Sydney.

A more sensitive indicator of income of low socio-economic families is likely to be indicative low disposable household income, as it reflects minimum wage earnings and welfare payments and real food buying power [19, 46]. It is not location dependent as it is based on national figures. Indicative low disposable household income was much lower than median gross household income in five of the six locations surveyed; the exception being the most disadvantaged area in Sydney. In these five areas, low income households spent on average 34% of disposable income on the current diet, but would spend a lesser proportion (28%) of disposable income on a healthy diet. In the area of most disadvantage in Sydney, both diets would be slightly more affordable, but still indicative of food stress. In both cities, low-income families need to commit more than 30% of their disposable income to purchase their current diet. So how are they managing?

Available data suggest not very well. At least 3.7% of Australian households report having run out of food in the previous 12 months and not being able to afford to buy more [10, 47]. This proportion is much higher among some groups, with this proxy measure of food insecurity affecting more than one in five Aboriginal and Torres Strait Islander people in Australia (22%), around 11% of people who are unemployed, and 16% of rental households [10]. Given this, reports of increasing reliance on food aid in Australia are not surprising [48]. However, while this study confirms the findings of other food pricing surveys in Australia that lower socioeconomic households need to spend a higher proportion of their income to procure healthy diets than other Australians [15, 49], it shows that the cost of the recommended (healthy) diet is lower than the current diet. This suggests that there is a need to better promote the potential cost savings, and health benefits, of shifting diets towards the recommendations of the Australian Dietary Guidelines [4].

Estimates of the affordability of the healthy diet assessed in this study were similar to those of ‘healthy’ diets in other community-based studies, although such diets were not necessarily consistent with dietary recommendations [15]. Previous studies reported ‘healthier’ diets cost between 28 and 40% of the disposable income of the lowest income families, compared with 20% for families on the average income [8], and up to 48% if a more limited number of foods was selected on the basis of their effects on environmental sustainability also [12].

### Differences between cities

Consistent with previous studies, the cost of food tended to be higher in areas of higher socioeconomic status in both cities [50], and this held in comparison between the two cities, with the cost of food being on average 4% higher in Canberra too. However, in Sydney this socioeconomic gradient was most marked for unhealthy diets: the relative difference in cost of unhealthy compared to healthy diets was greatest in low socioeconomic areas – potentially driving increased consumption of unhealthy diets among low income families in these areas. In contrast, both the costs of, and the price differential between, the current and the healthy diets were ‘flatter’ across different areas of socioeconomic status in Canberra, varying by only 1–2%.

The underlying causes of such differences between the cities are not clear. As most food manufacturing centres are located on the east coast of Australia, differences in food prices between the two cities may reflect additional transport costs to Canberra. However, while both cities have over 60% of people aged 15 years and over in full-time employment, the median gross household income in Canberra is much higher than in Sydney [21, 22], so price differences may also reflect consumers’ capacity to pay. While food prices are higher in Canberra than Sydney, the costs of other living expenses, such as housing rental/purchase, are much higher in Sydney [51]. Among low income groups, the money spent on food may be perceived to be more flexible than other household budget expenditure [52, 53]. Future studies could consider the impact of other factors contributing to the cost of living that may influence food purchasing behavior.

### Food costs: perceptions and interventions

Perception that healthy food is less affordable than unhealthy food may also affect food choice and consumption [54], particularly in low income families [55]. A recent study found unhealthy foods are advertised as reduced priced specials twice as often as healthy foods, which may contribute to inaccurate perceptions about the relative costs [56]. Study findings also support efforts to address low incomes of vulnerable households to improve food security in Australia [57].

As noted above, implications of this study suggest greater effort is required to promote healthy diets, including that healthy diets can be less expensive than current diets. Targeted taxes and subsidies on healthy foods can influence consumer food choices and be used to incentivise healthy eating at the population level [58, 59]. Targeted subsidies on fruit and vegetables appear to be most effective at increasing consumption [60, 61]. Other strategies that could help improve affordability of healthy foods among lower income groups include buying local fresh produce in season, seeking healthy foods on special offer; buying irregular-shaped, discounted produce, and choosing healthy generic ‘home brand’ products. Known barriers to healthy choices such as low food literacy and cooking skills also need to be addressed [4, 62]. Factors such as convenience and taste preferences should be explored further. However, the mounting evidence that current food environments are not conducive to supporting healthier choices [63] and the insights provided by this study, support the notion that better monitoring and surveillance of diet cost and affordability are urgently needed.

As healthy diets are not being consumed currently in Australia, it is critical not to create further barriers to healthy eating, such as by extending the base of the Goods and Services Tax to apply to basic healthy foods and increasing their cost [19]. The introduction of health levies on popular discretionary items may also help [58]; examples include volumetric tax on alcoholic drinks [64] and a 20% tax on sugary drinks [65]. This study has identified that, if purchasing patterns were unchanged, the latter strategy would cost the average family in Canberra or Sydney A$1.38 per fortnight. Despite critics suggesting that such interventions would be regressive, health levies on sugary drinks have been shown to be most beneficial for low income groups [66].

### Limitations

There are several methodological limitations in the application of the Healthy Diets ASAP methods as reported in publication of the protocol [20], including assumptions such as the food being shared equitably by members of the household and that there is no home food production and no wastage. No adjustments were made for costs such as transport, time, cooking equipment and utilities. As these costs apply to both diets, assessment of the price differential between the two can help control for some of these hidden costs; however the effect of these would increase actual costs of both diets and decrease their affordability [67].

The true costs of the recommended diet for the whole population would also likely be higher than reported, as this diet is modelled on the Foundation Diet prescribed for the shortest and least active members in each age and gender group according to NHMRC methods [4, 68]; hence it underestimates the requirements of taller, more active and overweight/obese individuals.

The true costs of the current diet would also likely be higher than reported, as no adjustments were made to account for the under-reporting in the AHS 2011–12 [69] or for the greater proportion of pre-prepared ‘convenience’ items in the current diet compared with the healthy diet. However, the similarity of the total energy of the current and healthy diets further highlights the comparability of these diet modellings used in the Healthy Diet ASAP.

The sample size potentially restricts analysis and generalisability of the findings. As with all field survey data collection methods, it takes time and resources to collect and analyze data. These processes can be streamlined through application of technologies such as electronic data collection and/or data scraping which are currently being developed [70].

## Conclusions

In reporting the first application of Healthy Diets ASAP methods to compare cost and affordability of current and healthy diets in two cities, this study confirms that standardized assessment of these metrics is an important aspect of monitoring and surveillance efforts to inform fiscal and health policy actions to support improved nutrition and diet-related health. The method could be adapted readily to investigate diet cost and affordability in other countries.

This study provides evidence that a healthy diet, consistent with national dietary guidelines, can be less expensive than current diets. More effort is needed to counter perceptions that healthy diets are more expensive than unhealthy diets. However, while less expensive, healthy diets may still not be affordable for families with low household income, who are more vulnerable to food stress and food insecurity. Policy actions to further decrease the cost of healthy foods, including relative to unhealthy choices, are needed urgently.

## Supplementary information

**Additional file 1: Supplementary information file 1.** Details of the current (unhealthy) and healthy (recommended) diets: total energy of food baskets per representative household per day (kJ/day) and foods comprising diet baskets per representative household per fortnight. **Supplementary information file 2.** A. Median income data from the 2011 Census, ABS Community Profiles of SA2 areas for the six SA2 locations in Sydney, NSW. **B.** Median income data from the 2011 Census, ABS Community Profiles of SA2 areas for the six SA2 locations in Canberra, ACT. **Supplementary information file 3.** Calculations of low (minimum) household income data from welfare data.

## Data Availability

All data generated or analysed during this study are included in this published article and its three supplementary information files.
