# Supplementary material for: A tale of two cities: the cost, price-differential and affordability of current and healthy diets in Sydney and Canberra, Australia
Source: Int J Behav Nutr Phys Act. 2020 Jun 22;17:80. doi: 10.1186/s12966-020-00981-0 (PMC7309977; doi:10.1186/s12966-020-00981-0)
Supplement: Supplementary file 1 — Additional file 1: Supplementary information file 1. Details of the current (unhealthy) and healthy (recommended) diets: total energy of food baskets per representative household per day (kJ/day) and foods comprising diet baskets per representative household per fortnight. Supplementary information file 2. A. Median income data from the 2011 Census, ABS Community Profiles of SA2 areas for the six SA2 locations in Sydney, NSW. B. Median income data from the 2011 Census, ABS Community Profiles of SA2 areas for the six SA2 locations in Canberra, ACT. Supplementary information file 3. Calculations of low (minimum) household income data from welfare data. [file 12966_2020_981_MOESM1_ESM.docx]

**Supplementary information file 1: Details of the current (unhealthy) and healthy (recommended) diets: total energy of food baskets per representative household per day (kJ/day) and foods comprising diet baskets per representative household per fortnight.**

**Supplementary information file 2: A. Median income data from the 2011 Census, ABS Community Profiles of SA2 areas for the six SA2 locations in Sydney, NSW**

**Names of suburbs de-identified**

|  | SA2 - **NSW** | Median PERSONAL income (excluding govt allowances/ pensions)  ($/year) | Average HH size (persons) | Median total HH income ($/weekly)*** | Median total HH income x1.111 ($/week)  (Includes Wage Price index increase of 11.1% from Sep 2011 – Sep 2015) | Median total Family income ($/weekly) | Median total Family income x1.111 ($/week)  (Includes Wage Price index increase of 11.1% from Sep 2011 – Sep 2015) | Median rent ($/week) | Median mortgage repayments ($/month) |
| --- | --- | --- | --- | --- | --- | --- | --- | --- | --- |
| Q1 | Area A (SEIFA 806-972) | $34,002 | 3.0 | $858 | $953 | $897 | $996 | $295 | $1,517 |
| Q1 | Area B (SEIFA 772-963 | $37,805 | 3.1 | $824 | $915 | $943 | $1,047 | $225 | $1,790 |
| Q3 | Area C (SEIFA 927-1035) | $47,926 | 3.3 | $1,518 | $1,686 | $1,544 | $1,715 | $350 | $2,000 |
| Q3 | Area D (SEIFA 770-1053) | $45,748 | 2.8 | $1,266 | $1,406 | $1,440 | $1,600 | $330 | $2,000 |
| Q5 | Area E (SEIFA 1024-1153 | $61,301 | 2.4 | $2,039 | $2,265 | $2,540 | $2,822 | $470 | $2,817 |
| Q5 | Area F (SEIFA 1000-1204) | $48,403 | 3.0 | $2,037 | $2,263 | $2,275 | $2,527 | $650 | $2,900 |

**B. Median income data from the 2011 Census, ABS Community Profiles of SA2 areas for the six SA2 locations in Canberra, ACT**

**Names of suburbs de-identified**

|  | SA2 - **ACT** | Median PERSONAL income (excluding govt allowances/ pensions)  ($/year) | Average HH size (persons) | Median total HH income ($/weekly)*** | Median total HH income x1.111 ($/week)  (Adjusted to include Wage Price index increase of 11.1% from Sep 2011 – Sep 2015) | Median total Family income ($/weekly) | Median total Family income x1.111 ($/week)  (Adjusted to include Wage Price index increase of 11.1% from Sep 2011 – Sep 2015 | Median rent ($/week) | Median mortgage repayments ($/month) |
| --- | --- | --- | --- | --- | --- | --- | --- | --- | --- |
| Q1 | Area A (SEIFA range 1018-1112) | $56,240 | 2.8 | $1,861 | $2,068 | $2,035 | $2,261 | $380 | $1,950 |
| Q1 | Area B (SEIFA range 801-1164) | $59,906 | 2.0 | $1,344 | $1,493 | $2,515 | $2,794 | $150 (high prop of flats) | $2,019 |
| Q3 | Area C (SEIFA range 1044-1155) | $56,988 | 2.7 | $1,996 | $2,218 | $2,310 | $2,566 | $390 | $2,000 |
| Q3 | Area D (SEIFA range 1006-1151) | $58,011 | 2.8 | $1,976 | $2,195 | $2,210 | $2,455 | $370 | $2,080 |
| Q5 | Area E (SEIFA range 1061-1197) | $63,910 | 3.1 | $2,583 | $2,870 | $2,671 | $2,967 | $423 | $2,167 |
| Q5 | Area F (SEIFA range 1153-1208) | $78,024 | 2.4 | $2,935 | $3,261 | $3,556 | $3,951 | $580 | $2,500 |

**Notes for Supplementary information file 2:**

*** Median income data from the 2011 Census, ABS Community Profiles of SA2 areas for the SA2 locations in Canberra, ACT and Sydney, NSW.

Median total household income is applicable to occupied private dwellings. It excludes households where at least one member aged 15 years and over did not state an income and households where at least one member aged 15 years and over was temporarily absent on Census Night. It excludes 'Visitors only' and 'Other non-classifiable' households.

Total Household Income (weekly): <http://www.abs.gov.au/websitedbs/censushome.nsf/home/cpexplanatorynotes?opendocument&navpos=230>

Household income is calculated by summing the individual incomes reported by all household members aged 15 years and over. The Census collects individual income in ranges. Before they can be summed, a specific dollar amount needs to be allocated to each person. Median incomes for each range (derived using data from the Survey of Income and Housing) are used for this purpose.

Household income is not calculated where a household member aged 15 years and over did not state their income, or was temporarily absent. These households are coded to the Partial income stated category.

Total Personal Income (weekly): This variable indicates the total weekly income that is received and includes pensions and allowances. People are not asked to state their exact income, only to indicate the range into which their income falls. Applicable only to persons aged 15 years and over.

Median total family income is applicable to families in family households. It excludes families where at least one member aged 15 years and over did not state an income and families where at least one member aged 15 years and over was temporarily absent on Census Night.

Average household size is applicable to number of persons usually resident in occupied private dwellings. It includes partners, children, and co-tenants (in group households) who were temporarily absent on Census Night. A maximum of three temporary absentees can be counted in each household. It excludes 'Visitors only' and 'Other non-classifiable' households.

Wage price index is calculated quarterly. I have taken the % change over the previous year, see below, between Sep 2011 and Sep 2015 and added it to result in an 11.1% increase of wages over that period.

- Sep 2011-Sep 2012 3.7%
- Sep 2012-Sep 2013 2.6%
- Sep 2013-Sep 2014 2.5%
- Sep 2014-Sep 2015 2.3%

Limitations - The median HH income includes all types of families and households, and does not relate to the representative household of the Healthy Diets ASAP method.

**Supplementary information file 3: Calculations of low (minimum) household income data from welfare data**
